# Sugar in the Liver

**Published:** 1858-01

**Authors:** 


### ARTICLE VI.

### *Sugar in the Liver.*

WE have heretofore called attention to the views of M. Bernard, in regard to the secretion of sugar by the liver, even where no sugar has been taken in the food. As he has recently developed those views which have been very generally adopted by the medical profession, we believe our readers will thank us for a succinct account of the present state of this physiological doctrine.

The first opinion advanced by the eminent French physiologist, was that sugar was directly formed in the liver from some of the elements of the blood. Subsequently, however, he was led by the results of some experiments to change that view, and he then pointed out the fact that there existed in the liver, a substance which was susceptible of conversion into sugar. Recently he has more fully examined the matter, and has given to the world the results of his inquiries in reference to the physical and chemical characters of this glucogenic matter, for so it has been called.

These characters approximate it very closely to the well known vegetable product, starch, especially after change has commenced in it. It is a neuter substance, without smell or taste, and communicates to the tongue the same sensation as starch. Like starch it is capable of being suspended in water, to which it communicates a deep opaline tint. Under the microscope it presents nothing characteristic. With iodine, it strikes a color varying from a deep violet blue, to a clear maroon red, rarely a decided blue. Ignited with lime, it gives forth no ammoniacal vapors, showing the absence of nitrogen. It does not reduce salts of copper dissolved in potash, nor does it undergo the alcoholic fermentation when mixed with yeast.

Its analogies with hydrated starch are still more distinctly shown in the reactions of this matter with ferments. Diastase, and all the analogous vegetable ferments, pancreatic juice, saliva, blood, &c., all act on this ferment, precisely as they do on starch, transforming it into sugar through an intermediate stage, in which the product resembles dextrine. Prolonged ebullition in mineral acids, properly diluted, produces the same result. As this change takes place, the glucogenic matter gradually loses its power of assuming a blue tint under the action of iodine. At the same time the opalescence of its solution or rather mixture with water, disappears, in consequence of its passing from a state of suspension to one of solution. It now acquires all the properties of sugar. It produces the yellow precipitate with Trommer's test, (salts of copper and potash ;) it undergoes alcoholic fermentation, and disengages carbonic acid under the influence of yeast. Spontaneously, it does not change into sugar, and resists, to a certain extent, the process of putrefaction. Roasting, the limited action of ferments, and of the mineral acids, change the glycogenic matter into a substance resembling dextrine.

The process of forming sugar in the liver, therefore, consists of two distinct stages. First, the living hepatic tissue forms the glucogenic matter, which is then transformed into sugar, by the action of a ferment. This ferment is brought into contact with the substance upon which it is to act by means of the circulation, the ferment being, indeed, the liquid blood itself. Bernard demonstrates the existence of two distinct circulations, one the ordinary slow nutritive circulation, the other, intermittent, variable, the super-activity of which coincides with the appearance of an increased quantity of sugar in the organ. This over excitation of the portal circulation occurs naturally during digestion, and then the formation of sugar goes on with greater rapidity. The same condition may be brought about artificially during the interval of digestion, and then the same transformation takes place.

In hybernating, or benumbed animals, the retardation of the circulation caused by diminished temperature, causes a diminution or disappearance of sugar in the liver, but the restoration of the circulation by heat is accompanied by a reappearance of the sugar. Indeed, the formation of sugar may be made to go on or cease, by simply subjecting the animal alternately to the action of heat and cold.

In hot blooded animals, similar changes can be brought about through the agency of the nervous system. M. Bernard has shown that when the spinal cord is divided below the origin of the phrenic nerves, the activity of the hepatic circulation is greatly diminished, so that after four or five hours, no sugar remains in the tissue of the liver, though that organ contains abundance of glucogenic matter. On the other hand, if the wound be inflicted in the region of the fourth ventricle, the abdominal circulation is much accelerated, and the contact of the glucogenic matter with its ferment greatly extended, so that the transformation becomes sufficiently rapid to give the animal diabetes, because more sugar is made than can be got rid of in the normal way, and it therefore is washed out through the kidneys.

All this resembles very closely what goes on in the seed of plants. Starch is formed therein by a vital process of the parent vegetable; then by the contact of diastase, sugar is formed, a process purely chemical. So in the liver, removed from the body and placed in the proper conditions of heat and moisture, the transformation goes on as long as any ferment remains to act upon the glucogenic matter. So too, the same successive transformations through dextrine to sugar take place in both instances.

Pelouze has corroborated Bernard's statement, and has still further extended this interesting analogy, by his recent researches. He has succeeded in transforming the glucogenic matter into xyloidine, (gun cotton,) in the same manner as when operating upon starch. The xyloidine thus obtained, has all the properties of that procured from the vegetable substance, explodes at 356° F., and has the same weight in proportion to the material employed in its manufacture. Still further, boiling dilute nitric acid, forms with this substance, as with starch, oxalic acid.

"The analysis of the glucogenic matter, purified by heat, and dried in the stove," says this eminent chemist, "gave me the following numbers:

| | |
|---|---|
| Carbon, . . . . . . . | 39.8 |
| Hydrogen, . . . . . . . | 6.1 |
| Oxygen, . . . . . . . | 54.1 |
| | 100.0 |

Corresponding to the formula,

$$C^{12}\ H^{12}\ O^{12}.$$

"The composition of vegetable starch, placed in the same conditions, namely, having been treated with potassa, and afterwards dried at 212° F., corresponds with the formula,

$$C^{12}\ C^{11}\ H^{11}."$$

It, therefore, corresponds with the other members of the organic group to which starch belongs, in containing carbon combined with hydrogen and oxygen in the proportions of water.

Still further in corroboration of the views of Bernard, we may quote the experiments of Dr. Harley, published in the British and Foreign Medico-Chirurgical Review, for July. That gentleman ascertained that by stimulating the portal circulation of animals, by injecting stimulants into the portal vein, he could always produce excessive formation of sugar, manifested by a discharge of saccharine urine. He also found that he could excite diabetes in himself by taking certain stimulating articles of food.

On the other hand, Figuier, that indomitable opponent of Bernard's views, has published an account of some experiments, to prove that sugar is not found in the liver after death. He objects to the process of Bernard, for freeing the liver to be experimented on from glucose, and strangely misrepresents his views by calling the post-mortem formation of sugar, a posthumous secretion.

He says that Bernard's process for cleansing the liver, does not thoroughly wash away the sugar. He says that after thoroughly washing the pulp of a sheep's liver by decantation, till there remained nothing but a colorless pulp, no sugar was formed after twenty-four hours. He further states, that on washing a sheep's liver by a strong current of water, after Bernard's plan, but for a longer time, he still found sugar in the tissue, and that upon laying a liver so treated aside for twenty-four hours, the sugar does not increase. He, therefore, infers that Bernard's washing was insufficient to get rid of the sugar. He further goes on to say that he has succeeded in completely freeing from sugar, the liver of a horse, and that such a liver, once thoroughly freed from glucose, cannot reproduce it.

"In a fresh memoir on the glucogenic power of the liver," says he, "I shall very shortly have the honor of communicating to the Academy the result of some experiments on the glucogenic matter which exists in the liver, according to M. Cl. Bernard; and which, according to my views, is nothing but the product of the decomposition, by potassa, of *albuminose*—an organic product, the existence of which, in the liver, I have shown; and which was studied and described in a former memoir. I shall prove that this *glucogenic matter* is formed with most of the albuminoid substances, and may be obtained by operating with the albumen of eggs, precipitated by alcohol, redissolved in water, and treated with boiling caustic potassa. I shall likewise endeavor to show the chemical difference existing between the sugar contained in the liver, and that found in the vena portæ, and in the general circulation of animals fed on an exclusive meat diet."

It is evident that M. Figuier is merely skirmishing about the outposts of Bernard's theory. Even supposing him fully to establish all that he asserts touching the cleansing of the liver, he has only exhibited an error in the assertion of post-mortem changes, and has not cast any doubt upon the actions said to take place during life. Besides, it may be objected to his operations, that if Bernard has washed too little, he has, unquestionably, washed too much, and swept away, not only the sugar already formed, but the ferment which might have produced more from the glucogenic matter still present in the liver. The experiment on the pulp of the sheep's liver, goes even farther than this, since it must have washed away the glucogenic matter itself, which is, as we have just seen, suspensible in water.

As for his assertion about *albuminose,* that is already settled in advance, by what we have quoted from Pelouze. That chemist's well known accuracy, and the very decided results which he has obtained, completely upset the foreshadowed albuminose theory.

There is, however, still "another Richmond in the field" in the shape of M. A. Sanson, chief of the chemical department, at the veterinary school at Toulouse. That gentleman has asserted that he has obtained something analogous to dextrine, convertible into glucose, in short, Bernard's glucogenic matter, from other tissues of the body besides the liver, from the spleen, the kidneys, the lungs, and the muscles, a substance which he traces in every instance to the food, through the medium of the blood.

Here, however, he too, encounters Pelouze, who says he had the same idea, and examined these different tissues, to detect the glucogenic matter. He obtained, from the lungs, a substance, which, at first sight, presented the external characters of the glucogenic matter, forming like it, a whitish, flocculent precipitate. Here, however, the analogy stopped. It was impossible to make sugar out of it. Pelouze thinks it a modification of albumen, probably Mulder's tritoxyd of protein.

Sanson, however, is out in another paper, through which we have not the space to follow him. We, therefore, state his conclusions:

"1. In blood left undisturbed for forty-eight hours, there exists a fermentable sugar, which could not be found at the moment of its extraction from the vessel.

2. As it is impossible to admit a vital influence which has secreted it, we must acknowledge that it can only have been developed by the same means which give rise to it in the vegetable economy, namely, the action of diastase upon dextrine.

3. The experiment which demonstrates it, comes in support of the facts which I have previously mentioned, as to the presence, in the blood and all the tissues, of a glucogenic matter analogous to dextrine.

4. These facts prove, as I have already established, that the dextrine of the blood has its source, in herbivorous animals, in the action of the ptyaline on the amyloid principles of their aliments, and among the carnivora, in the flesh which they eat, and in which it is found already formed.

5. Finally, the liver does not, in either case, secrete either sugar or a glucogenic matter, but it merely serves, as well as the whole course of the other organs, to establish the contact of the dextrine and diastase, which contact is here only more prolonged, in consequence of the slacking of the rapidity of the circulation in the hepatic tissue."

In looking over these conclusions, we find the second a most complete example of the *non sequitur*. Why it is impossible to admit a vital influence to have secreted a substance found in the blood ; and if so why it is necessary to suppose that it reached that liquid in the manner specified, we confess ourselves unable to imagine. For the rest, we find nothing new in the whole paper, excepting the remarkable statement concerning the formation of sugar in blood, after standing a length of time. This is curious, and if established by other observers, may lead to interesting results, though there is nothing in it to overthrow Bernard's theory. We are distinctly told by that eminent physiologist, that the glucogenic matter undergoes the ordinary changes of starch, dextrine being always the intermediate stage between it and sugar. Now it is only necessary to suppose that some of this dextrine has passed into the circulation, before it could be converted into sugar, and the blood being a ferment to it, as well as to the glucogenic matter, the complete transformation takes place in that fluid.

As for this writer's other assertions, they have already been sufficiently disproved, as any one may satisfy himself by examining our previous article upon this subject.

We shall continue, from time to time, to keep our readers apprised of the progress of this interesting discussion, until the principles involved are fully established.

